# Enhanced Cytotoxic Effects in Human Oral Squamous Cell Carcinoma Cells Treated with Combined Methyltransferase Inhibitors and Histone Deacetylase Inhibitors

**DOI:** 10.3390/biomedicines10040763

**Published:** 2022-03-24

**Authors:** Ryosuke Ushio, Miki Hiroi, Ari Matsumoto, Kazumasa Mori, Nobuharu Yamamoto, Yoshihiro Ohmori

**Affiliations:** 1 Division of Oral and Maxillofacial Surgery, Department of Diagnostic and Therapeutic Sciences, Meikai University School of Dentistry, 1-1 Keyakidai, Sakado 350-0283, Japan; r-ushio-007@dent.meikai.ac.jp (R.U.); matsuari@dent.meikai.ac.jp (A.M.); kazu-mori@dent.meikai.ac.jp (K.M.); nyamamot@dent.meikai.ac.jp (N.Y.); 2 Division of Microbiology and Immunology, Department of Oral Biology and Tissue Engineering, Meikai University School of Dentistry, 1-1 Keyakidai, Sakado 350-0283, Japan; mikih@dent.meikai.ac.jp

**Keywords:** methyltransferase inhibitors, histone deacetylase inhibitors, epigenomic therapy, oral squamous cell carcinoma cells, cell viability, apoptosis, cell cycle arrest, γH2A.X

## Abstract

Combined treatment of human oral squamous cell carcinoma (OSCCs) with DNA methyltransferase inhibitors (DNMTis), histone methyltransferase inhibitors (HMTis), and histone deacetylase inhibitors (HDACis), and the molecular mechanisms underlying their anticancer effects, have not been fully elucidated. Herein, we investigated the cytotoxic effects of combined DNMTis (5-Aza-deoxycytidine: 5-Aza-dC, RG108), HMTis (3-deazaneplanocin A: DZNep), and HDACis (trichostatin A: TSA) treatment on human OSCC cells and explored their molecular mechanisms. Combined 5-Aza-dC, or RG108, and TSA treatment significantly decreased HSC-2 and Ca9-22 cell viability. Combinatorial DZNep and TSA treatment also decreased Ca9-22 cell viability. Although caspase 3/7 activation was not observed in HSC-2 cells following combined treatment, caspase activity was significantly increased in Ca9-22 cells treated with DZNep and TSA. Moreover, combined treatment with 5-Aza-dC, RG108, and TSA increased the proportion of HSC-2 and Ca9-22 cells in the S and G2/M phases. Meanwhile, increased phosphorylation of the histone variant H2A.X, a marker of double-stranded DNA breaks, was observed in both cells after combination treatment. Hence, the decreased viability induced by combined treatment with epigenomic inhibitors results from apoptosis and cell cycle arrest in S and G2/M phases. Thus, epigenomic therapy comprising combined low concentrations of DNMTi, HMTi, and HDACi is effective against OSCC.

## 1. Introduction

Carcinogenesis is caused by the accumulation of genetic abnormalities, which are broadly classified as genomic or epigenomic [1]. Genomic abnormalities include gene mutations and chromosomal translocations, while epigenomic abnormalities are caused by irregular DNA methylation, histone protein methylation, and acetylation [1,2,3]. Epigenomic abnormalities alter higher-order chromatin structures, resulting in abnormal gene expression [2,3]. In general, DNA methylation acts as a transcriptional repressor while histone acetylation acts as a transcriptional activator. Importantly, the epigenomic status of many cancers differs from that of normal cells [2,3]. For this reason, epigenomic drugs that target and regulate the epigenome have been developed and have demonstrated therapeutic efficacy against various malignancies [4].

DNA methylation is caused when DNA methyltransferase (DNMT) introduces a methyl group onto cytosine residues in CpG dinucleotide sequences [5]. High methylation at CpG islands occurs in the promoter regions of tumor suppressor genes, such as retinoblastoma tumor suppressor gene (*RB1*), cyclin-dependent kinase inhibitor 2A/p16^INK4a^ (*CDKN2A*), and breast cancer susceptibility gene (*BRCA1*) [2]. When DNA methylation occurs in the promoter regions of these tumor suppressor genes, transcription factor binding is inhibited and gene expression is suppressed by methylated CpG-binding proteins that interact with corepressor complexes [6]. Hence, restoring tumor suppressor gene expression, following suppression by epigenomic abnormalities, has been adopted as a strategy for cancer therapy. Indeed, several DNA methylation inhibitors have been developed using this strategy [4]. For instance, 5-Aza-2′-deoxycytidine (5-Aza-dc), a nucleoside DNMT inhibitor (DNMTi), is incorporated into the DNA during replication and irreversibly binds to DNMT1, thereby inhibiting its activity and inducing demethylation. [7]. RG108, a novel non-nucleoside small molecule DNMTi, has low cytotoxicity, and demethylates repress the tumor suppressor genes by inhibiting the DNMT active site [8]. However, the antitumor effects of RG108 on oral squamous cell carcinoma cells (OSCCs) and the concomitant effects of epigenomic drugs have not yet been clarified.

In addition to DNA methylation, post-translational modifications, such as methylation and acetylation of lysine residues at the N-termini of histone proteins, are involved in gene regulation [9]. Nucleosomes are composed of two molecules each of the core histones H2A, H2B, H3, and H4 that form an octamer surrounded by 146 bp of DNA [9]. In general, histone acetylation loosens the bonds between DNA and histones, resulting in an open and active chromatin state. In contrast, histone methylation has diverse effects such as gene activation or repression, depending on the position of the modified lysine residue [9]. Enhancer of zeste homolog 2 (EZH2), a histone methyltransferase (HMT) [10], induces trimethylation of histone H3 lysine 27 (H3K27me3), which suppresses gene expression [11]. EZH2 overexpression occurs in many solid tumors with advanced and metastatic phenotypes, such as prostate and breast cancers [12,13,14]. Meanwhile, the histone methyltransferase activity of EZH2 is inhibited by 3-deazaneplanocin A (DZNep), an HMT inhibitor (HMTi) [15]. Although the effects of DZNep have been reported for several solid tumors, including OSCCs [16,17], its effects, in combination with DNMTis, have not been clarified.

Histone acetylation refers to the addition of acetyl groups to lysine residues in histone H3 and H4 by histone acetyltransferases (HATs), resulting in a transcriptionally active chromatin state [18]. The addition of acetyl group effectively neutralizes the positive charge on lysine, thereby causing the negatively charged DNA to be repelled, while also weakening histone binding to DNA and promoting transcription factor binding [19]. Subsequently, histone deacetylases (HDACs), which are abundant in transcriptional repressor complexes, are recruited to methylated DNA regions, where they deacetylate histones, thereby reestablishing their positive charge, causing the chromatin structure to condense, and gene expression to be suppressed [20,21]. Additionally, HDACs regulate non-histone proteins involved in DNA replication and damage repair. Inhibiting HDACs suppresses DNA damage repair and induces cancer cell death [22]. Therefore, HDAC inhibitors (HDACis) are attracting attention as anticancer drugs [23,24]. For example, Trichostatin A (TSA), a potent HDACi that has been extensively applied in laboratory research [25,26], alters transcription by reorganizing the chromatin and changing the protein conformations of the transcription factor complexes [27]. Indeed, the effects elicited by HDACis, such as TSA, on cell cycle arrest, apoptosis, and gene expression have been widely reported in head and neck squamous cell carcinoma cells, including OSCCs [28,29,30,31,32,33]. However, although their antitumor effects have been clearly demonstrated in preclinical studies for hematologic tumors, monotherapy with HDACi offers only limited efficacy for solid tumors [34]. Therefore, a combination therapy of HDACis with other anticancer and epigenomic agents, including DNMTis, has been investigated in many solid tumors to enhance antitumor efficacy [35,36,37,38,39,40,41,42,43].

The efficacy of combination therapy with epigenomic drugs has been investigated in numerous malignancies [4,21,44,45,46]. In particular, the efficacy of combined DNMTi (5-Ada-dC) and HDACi therapy has been demonstrated in OSCC cells [41,42,43]. However, the efficacy of a combination treatment with RG108 [8], a novel DNMTi—and HDACi or DZNep [15], an HMTi—and HDACi, or a triple treatment with DNMTi, HMTi, and HDACi has not yet been reported. Accordingly, the purpose of the current study is to investigate the combined cytotoxic effects of these epigenomic drugs on OSCC cells and to explore their underlying molecular mechanisms.

Herein, we demonstrate that combining low concentrations of DNMTi, HMTi, and HDACi significantly decreases OSCC viability via a mechanism that is not solely based on induction of apoptosis. Rather, this combinatorial therapy appears to arrest cell cycle development in the S and G2/M phases via induction of DNA damage. Taken together, the results of this study demonstrate the efficacy of combining low concentrations of epigenetic drugs to target OSCC cells, while also contributing to the current understanding regarding the molecular mechanisms underlying combined epigenetic therapy.

## 2. Materials and Methods

### 2.1. Reagents

The 5-Aza-dC was obtained from Selleck Chemicals (Houston, TX, USA). RG108 and DZNep were obtained from Tocris Bioscience (Bristol, UK). TSA was obtained from Merck (Darmstadt, Germany). Dimethyl sulfoxide (DMSO), propidium iodide (PI), protease inhibitor cocktail, and phosphatase inhibitor cocktail were purchased from Sigma-Aldrich (St. Louis, MO, USA). Cell Counting Kit-8 was obtained from Dojin (Kumamoto, Japan). The Caspase-Glo 3/7 Assay System was purchased from Promega (Madison, WI, USA). The 5-Aza-dC, RG108, and TSA were dissolved in DMSO, while DZNep was dissolved in distilled water. All compounds were stored at −20 °C until use.

### 2.2. Cell Culture

The human OSCC cell lines Ca9-22 (cat#: JCRB0625) and HSC-2 (cat#: JCRB0622) were obtained from the Japanese Collection of Research Bioresources (JCRB) cell bank (Osaka, Japan). Ca9-22 cells were isolated from a primary gingival carcinoma [47] and HSC-2 cells were isolated from cervical lymph node metastatic SCC derived from the floor of the oral cavity [48]. These cells are considered to be highly differentiated [47,48]. Cells were grown in RPMI-1640 containing 10% fetal bovine serum (FBS; Bio West, Miami, FL, USA) and 1% penicillin/streptomycin sulfate (Thermo Fisher Scientific, Waltham, MA, USA), hereafter designated as complete medium. For passaging, cells were washed with phosphate-buffered saline (PBS; Thermo Fisher Scientific). Single-cell suspensions were obtained using 0.25% trypsin/0.01% EDTA (Thermo Fisher Scientific) and adjusted to the desired cell numbers for experiments.

### 2.3. Measurement of Cell Viability

Cells were seeded in 96-well flat-bottom plates (Becton Dickinson Labware, Franklin Lakes, NJ, USA) at a density of 3 × 10^3^ cells/well and cultured in complete medium for 24 h at 37 °C and 5% CO_2_. The cells were then treated with DNMTi (5-Aza-dc, RG108), HMTi (DZNep), and HDACi (TSA) for 24, 48, 72, and 96 h, respectively. The control group was treated with the same amount of DMSO or distilled water. After inhibitor treatment, 10 μL of WST-8 reagent from the Cell Counting Kit-8 (Dojin) was added to each well and incubated for 2 h at 37 °C. The optical density at 450 nm was then measured using a microplate reader (Multiskan Bichromatic; Labsystems Diagnostics, Helsinki, Finland). Cell viability was calculated as the percent viability (%) of the inhibitor group compared to the control group.

### 2.4. Measurement of Caspase Activity

Caspase activity was measured using the Caspase-Glo 3/7 Assay System (Promega). Briefly, 3 × 10^3^ cells/well were seeded in 96-well white flat-bottomed plates (Thermo Fisher Scientific) and incubated in complete medium at 37 °C and 5% CO_2_ for 24 h. The cells were then treated with various inhibitors for 24 h. Next, 100 µL of culture supernatant was removed from each well and 100 µL of Caspase-Glo 3/7 Assay Reagent (caspase 3/7 substrate to which chemiluminescent luciferin was added) was added to each well. The mixture was incubated at 37 °C for 30 min. Chemiluminescence was measured using a luminometer (Centro XS3 LB960, BERTHOLD, Bad Wildbad, Germany). Caspase activity was expressed as the caspase activity of the inhibitor group relative to that of the control group.

### 2.5. Cell Cycle Analysis

Ca9-22 and HSC-2 cells were seeded in 6-cm dishes (Becton Dickinson Labware) at 3.6 × 10^5^ cells/well and 1.8 × 10^5^ cells/well, respectively. The cells were then incubated in complete medium for 24 h and subsequently treated with inhibitors for 48, 72, and 96 h. After incubation, cells were treated with 0.25% trypsin/0.01% EDTA, washed with ice-cold PBS, and fixed with 70% cold ethanol. The cells were incubated with 250 µg/mL RNase A (Nippon Gene, Tokyo, Japan) at 37 °C for 30 min, followed by 50 µg/mL PI at 4 °C for 30 min. For cell cycle analysis, PI-stained cells (1 × 10^4^ cells) were analyzed using a flow cytometer (Cell Sorter SH800, Sony, Tokyo, Japan). The percentage of cells at different stages of the cell cycle was analyzed using Cell Sorter Software Ver. 2.1 (Sony, Tokyo, Japan).

### 2.6. Preparation of Cell Extracts

Ca9-22 and HSC-2 cells were seeded in 10-cm dishes (Becton Dickinson Labware) at a density of 1 × 10^6^ cells/dish and 5 × 10^5^ cells/dish, respectively. The cells were then incubated in complete medium for 24 h, followed by incubation with inhibitors for 48 and 72 h. Next, the cells were washed with ice-cold PBS, collected using a cell scraper (Corning, Corning, NY, USA), and centrifuged at 2400× *g* for 3 min at 4 °C. The precipitated cells were incubated with 300 µL of IP kinase buffer (50 mM HEPES (pH 8.0), 150 mM NaCl, 2.5 mM EGTA, 1 mM EDTA, 1 mM dithiothreitol (DTT), 0.1% Tween 20, 10% glycerol, 1 mM phenylmethylsulfonyl fluoride (PMSF), 1% (*v*/*v*) protease inhibitor cocktail, 1% (*v*/*v*) phosphatase inhibitor cocktail, 1 mM NaF, and 1 mM NaVO_4_). The cell suspension was kept on ice for 10 min, lysed by ultrasonic disruption (Bioruptor UCW-310, Sonicbio, Kanagawa), and centrifuged at 12,000× *g* for 10 min. The supernatant was used as the total cellular extract and the protein concentration of the extracts was measured using the Bradford method [49] with protein dye reagent (Bio-Rad, Hercules, CA, USA).

### 2.7. Western Blotting Analysis

The cell extracts were mixed with 4× Laemmli Sample buffer (Bio-Rad), denatured at 95 °C for 5 min, and fractionated by Sodium dodecyl sulfate-polyacrylamide gel electrophoresis using 4–20% Mini-PROTEAN TGX Stain-Free Precast Gels (Bio-Rad). After electrophoresis, images of the total proteins in each lane were captured using a ChemiDoc MP imaging system (Bio-Rad). The proteins were then transferred to polyvinylidene fluoride (PVDF) membranes (Bio-Rad) using a semi-dry transfer system (Trans-Blot Turbo, Bio-Rad). After transfer, the PVDF membranes were blocked with 0.1% bovine serum albumin (BSA; Roche, Basel, Switzerland) in Tris-buffered saline (TBS)-Tween buffer (20 mM Tris-HCl (pH 7.4), 137.5 mM NaCl, and 0.1% Tween-20) for 1 h at 25 °C and incubated with anti-phospho-histone H2A.X (Ser139) rabbit monoclonal antibody (cat#9718; dilution: 1/1000; Cell Signaling Technology, Danvers, MA, USA) at 4 °C for 12 h. After washing with TBS-Tween buffer, the blots were incubated with horseradish peroxidase-conjugated donkey anti-rabbit IgG (dilution: 1/2000; GE Healthcare, Chicago, IL, USA) for 2 h and washed again with TBS-Tween buffer. The membranes were developed using West Pico Chemiluminescent Substrate (Pierce, Rockford, IL, USA). Chemiluminescent signals were captured using a ChemiDoc MP imaging system (Bio-Rad). The bands detected by the primary antibodies were normalized to the total protein and calculated as the fold expression relative to the control group.

### 2.8. Statistical Analysis

Statistical analyses were performed using GraphPad Prism (Version 9.1.0, GraphPad Software, San Diego, CA, USA). One-way analysis of variance (ANOVA) was used for comparisons among multiple groups of three or more. Dunnett’s method was used for multiple comparisons with the control group. Tukey’s method was used to compare each group. *p* < 0.05 was considered statistically significant.

## 3. Results

### 3.1. Effect of DNMTi on Cell Viability of OSCC Cells

**Figure 1:**
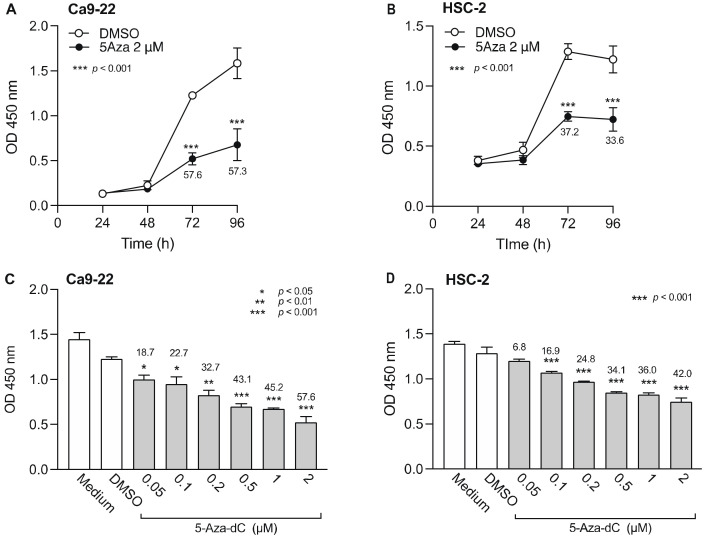
Effect of 5-Aza-dC, a DNMTi, on Ca9-22 and HSC-2 cell viability. Viability of Ca9-22 (**A**) and HSC-2 (**B**) cells following treatment with 5-Aza-dC (2 µM) or DMSO for the indicated periods of time. Viability of Ca9-22 (**C**) and HSC-2 (**D**) cells following treatment with various concentrations of 5-Aza-dC for 72 h. Data are presented as the mean ± SEM of three independent experiments. Significant differences were assessed by one-way ANOVA (* *p* < 0.05, ** *p* < 0.01, *** *p* < 0.001). The percent inhibition of cell viability is shown above bars.

To determine how DNMTi treatment time affects OSCC cells, 5-Aza-dC (2 µM) or DMSO was added to Ca9-22 and HSC-2 cells and cell viability was measured over time (Figure 1A,B). Ca9-22 and HSC-2 cell viability significantly decreased 72 h after addition of 5-Aza-dC. Next, to determine the minimum effective concentration of 5-Aza-dC, we added various concentrations of 5-Aza-dC and measured the cell viability after 72 h (Figure 1C,D). The 5-Aza-dC significantly decreased Ca9-22 and HSC-2 cell viability in a concentration-dependent manner. The minimum effective concentrations of 5-Aza-dC for Ca9-22 and HSC-2 cells were 0.05 µM and 0.1 µM, respectively. Thus, we selected 0.1 µM 5-Aza-dC for combination treatment with epigenomic drugs in subsequent experiments.

**Figure 2:**
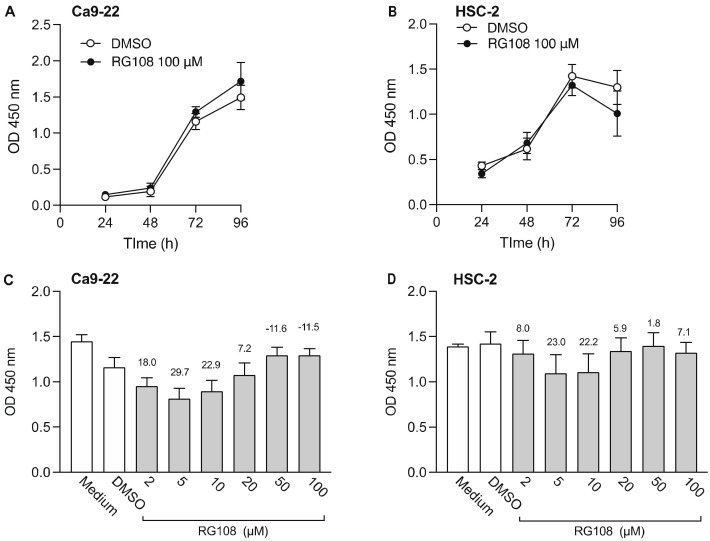
Effect of RG108, a DNMTi, on Ca9-22 and HSC-2 cell viability. Viability of Ca9-22 (**A**) and HSC-2 (**B**) following treatment with RG108 (100 µM) or DMSO for the indicated periods of time. Viability of Ca9-22 (**C**) and HSC-2 (**D**) cells following treatment with various concentrations of RG108 for 72 h. Data are presented as the mean ± SEM of three independent experiments. The percent inhibition of cell viability is shown above bars.

Next, we examined the effect of RG108, a non-nucleoside DNMTi, on the viability of Ca9-22 and HSC-2 cells (Figure 2). Results show that 100 µM RG108 did not significantly decrease cell viability from 24 to 96 h (Figure 2A,B). Therefore, we added various concentrations of RG108 and examined cell viability after 72 h. Low concentrations of RG108 (5 µM and 10 µM) tended to decrease Ca9-22 and HSC-2 cell viability. However, no significant difference was observed between the RG108 and DMSO treatments. In subsequent experiments of combined treatment with epigenomic drugs, we opted to use 5 µM RG108 as this concentration showed a tendency to decrease cell viability.

### 3.2. Effect of HMTi on Cell Viability of OSCC Cells

**Figure 3:**
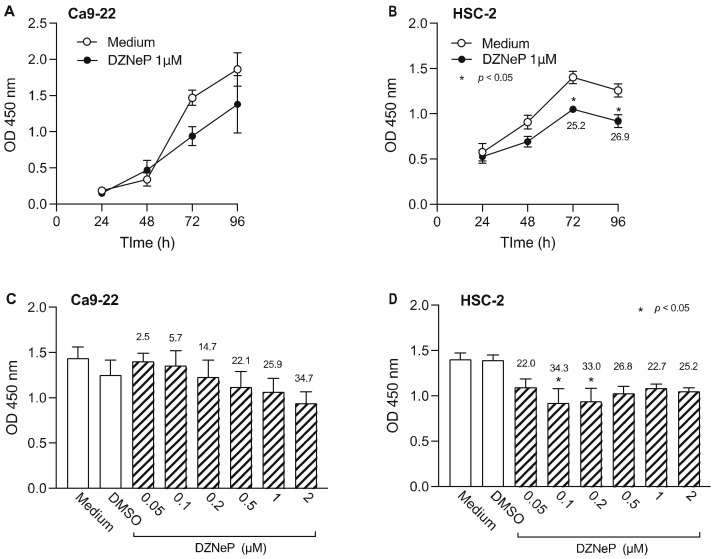
Effect of DZNep, an HMTi, on Ca9-22 and HSC-2 cell viability. Viability of Ca9-22 (**A**) and HSC-2 (**B**) following treatment with DZNep (1 µM) or DMSO for the indicated periods of time. Viability of Ca9-22 (**C**) or HSC-2 (**D**) cells following treatment with various concentrations of DZNep for 72 h. Data are presented as the mean ± SEM of three independent experiments. Significant differences were assessed by one-way ANOVA (* *p* < 0.05). The percent inhibition of cell viability is shown above bars.

Next, we examined the effect of DZNep, an HTMi, on Ca9-22 and HSC-2 cell viability (Figure 3). DZNep (1 µM) decreased the viability of Ca9-22 cells after 72 h, however the difference was not statistically significant (Figure 3A). In contrast, we observed significantly decreased viability of HSC-2 cells after 72 h (Figure 3B). We also examined the effect of various DZNep concentrations on cell viability and observed a concentration-dependent decrease in Ca9-22 cell viability, however, the decrease was not significant (Figure 3C). In contrast, 0.1 µM and 0.2 µM DZNep significantly decreased HSC-2 cell proliferation (Figure 3D). Based on these results, 0.2 µM DZNep was used in subsequent experiments.

### 3.3. Effect of HDACis on the Viability of OSCC Cells

**Figure 4:**
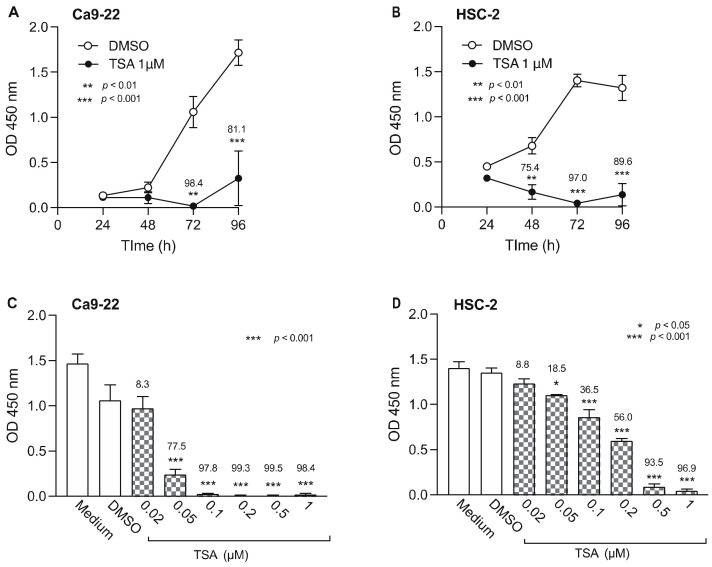
Effect of TSA, a HDACi, on Ca9-22 and HSC-2 cell viability. Viability of Ca9-22 (**A**) and HSC-2 (**B**) cells following treatment with TSA (1 µM) or DMSO for the indicated periods of time. Viability of Ca9-22 (**C**) and HSC-2 (**D**) cells following treatment with various concentrations of TSA for 72 h. Data are presented as the mean ± SEM of three independent experiments. Significant differences were assessed by one-way ANOVA (* *p* < 0.05, ** *p* < 0.01, *** *p* < 0.001). The percent inhibition of cell viability is shown above each bar.

We also examined the effect of TSA, an HDACi, on the viability of Ca9-22 and HSC-2 cells (Figure 4). TSA (1 µM) significantly decreased Ca9-22 cell viability after 72 h (Figure 4A) and HSC-2 cell viability after 48 h (Figure 4B). When we tested various concentrations of TSA, we observed a concentration-dependent decrease in Ca9-22 and HSC-2 cell viability beginning at 0.05 µM TSA in both cell lines (Figure 4C,D). Thus, 0.05 µM TSA was used for combination treatment with epigenetic drugs in subsequent experiments.

Taken together, these results indicate that treatment of Ca9-22 and HSC-2 cells with RG108 or DZNep alone resulted in marginal cytotoxic effects, while 5-Aza-dc and TSA alone induced significant levels of cytotoxicity.

### 3.4. Combined DNMTi, HMTi, and HDACi Treatment Reduces OSCC Cell Viability

**Figure 5:**
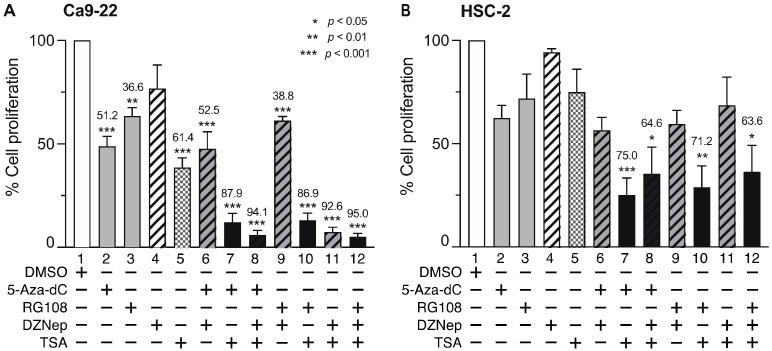
Effect of combined DNMTis, HMTis, and HDACis treatment on cell viability. Viability of Ca9-22 (**A**) and HSC-2 (**B**) cells following treatment with 5-Aza-dC (0.1 µM), RG108 (5 µM), DZNep (0.2 µM), or TSA (0.05 µM) alone or in combination for 72 h. Data are presented as the mean ± SEM of three independent experiments. Significant differences were assessed by one-way ANOVA (* *p* < 0.05, ** *p* < 0.01, *** *p* < 0.001). The percent inhibition of cell viability is shown above each bar.

We next examined the effect of combined treatment with the minimum effective concentrations of DNMTi, HMTi, and HDACi for 72 h on Ca9-22 and HSC-2 cell viability (Figure 5). In Ca9-22 cells, 5-Aza-dC or RG108 and TSA significantly decreased cell viability compared to treatment with each compound alone (Figure 5A, lanes 7 and 10). Combined treatment with DZNep and TSA also significantly decreased cell viability (Figure 5A, lane 11). Similarly, decreased Ca9-22 cell viability was observed after combined treatment with 5-Aza-dC or RG108 with DZNep and TSA (Figure 5A, lanes 8 and 12). However, combined treatment with either 5-Aza-dC or RG108 and DZNep induced minimal effects, compared to that observed following treatment with each compound alone (Figure 5A, lanes 6 and 9).

In HSC-2 cells, combined treatment with 5-Aza-dC or RG108 and TSA decreased cell viability (Figure 5B, lanes 7 and 10), however, the effect was weaker than that in Ca9-22 cells. Interestingly, combined treatment of DZNep and TSA (Figure 5B, lanes 11 and 12) did not further reduce the decreased viability observed in Ca9-22 cells. These results indicate that combined treatment with a DNMTi (5-Aza-dC, RG108) and an HDACi (TSA) at the minimum effective concentration significantly decreased Ca9-22 and HSC-2 cell viability compared to treatment with each compound alone. Additionally, combined treatment with DZNep and TSA also significantly decreased Ca9-22 cell viability.

### 3.5. DNMTis, HMTis, and HDACis Alter Caspase 3/7 Activity in OSCC Cells

**Figure 6:**
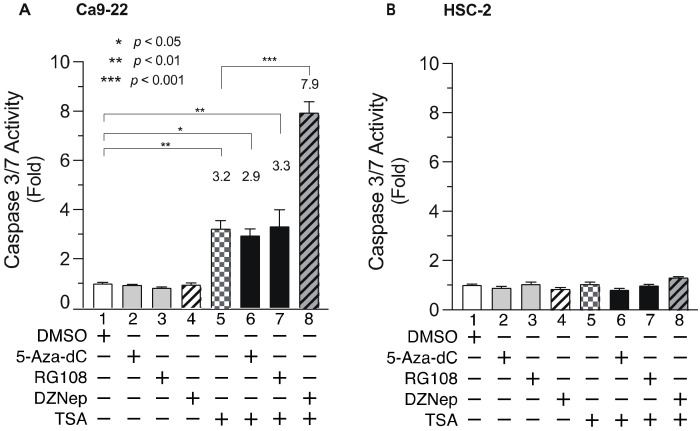
Caspase 3/7 activity in Ca9-22 and HSC-2 cells treated with DNMTis, HMTis, and HDACis. Caspase 3/7 activity levels in Ca9-22 (**A**) or HSC-2 (**B**) cells following treatment with 5-Aza-dC (0.1 µM), RG108 (5 µM), DZNep (0.2 µM), or TSA (0.05 µM) alone or in combination for 24 h. Caspase activity is expressed relative to activity in DMSO-treated cells. Data are presented as the mean ± SEM of three independent experiments. Significant differences were assessed by one-way ANOVA. (* *p* < 0.05, ** *p* < 0.01, *** *p* < 0.001). The fold induction of caspase activity is shown above each bar.

To elucidate the mechanism underlying the decreased cell viability following combined treatment with DNMTis, HMTis, and HDACis, we analyzed the activity of caspase 3/7 (Figure 6), which is an effector caspase that initiates apoptosis [50,51]. Ca9-22 cells were treated with various inhibitors for 24 h and caspase 3/7 activity was examined. Although single treatment with 5-Aza-dC, RG108, or DZNep did not elicit significant effects (Figure 6A, lanes 2, 3, and 4), TSA treatment alone increased caspase 3/7 activity by approximately 3-fold (Figure 6A, lane 5). Meanwhile, combined treatment with 5-Aza-dC or RG108 and TSA did not enhance caspase 3/7 activity (Figure 6A, lanes 6 and 7). In contrast, combined DZNep and TSA significantly enhanced caspase 3/7 activity in Ca9-22 cells (Figure 6A, lane 8). Moreover, no significant increase was observed in HSC-2 cells after treatment with epigenomic drugs alone or in combination (Figure 6B). These results indicate that the decreased cell viability after combined treatment with DNMTis, HMTis, and HDACis was not due to apoptosis alone.

### 3.6. DNMTis, HMTis, and HDACis Induce Cell Cycle Arrest in OSCC Cells

**Figure 7:**
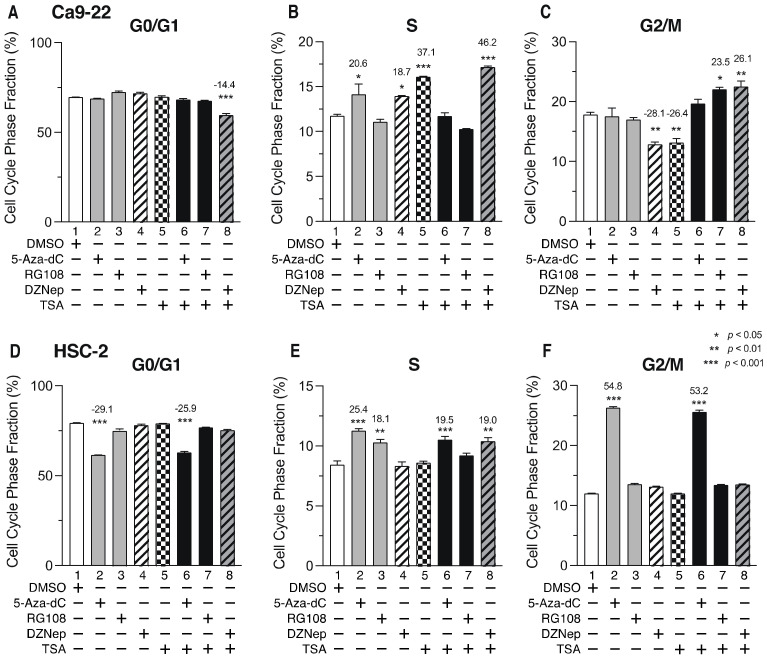
Proportion of Ca9-22 and HSC-2 cells in each phase of the cell cycle after treatment with DNMTis, HMTis, and HDACis. Proportion of Ca9-22 (**A**–**C**) or HSC-2 (**D**–**F**) cells in each stage of the cell cycle (G0/G1: (**A**,**D**); S: (**B**,**E**); G2/M: (**C**,**F**)) following treatment with 5-Aza-dC (0.1 µM), RG108 (5 µM), DZNep (0.2 µM), or TSA (0.05 µM) alone or in combination for 72 h. Data are presented as the mean ± SEM of three independent experiments. Significance of the differences in cell cycle phase between DMSO-treated and inhibitor-treated samples was assessed by one-way ANOVA (* *p* < 0.05, ** *p* < 0.01, *** *p* < 0.001). The percentage difference of cell cycle phase is shown above each bar.

To determine whether cell cycle arrest contributes to the decreased cell viability after combined treatment with epigenomic inhibitors, we examined the effects of DNMTi, HMTi, and HDACi on the cell cycle. Figure 7 shows the ratio of cells in each phase of the cell cycle 72 h after the addition of epigenetic drugs. Although cells in the G0/G1 phase in Ca9-22 cells were not increased by the inhibitors (Figure 7A), the proportion of cells in the S-phase was increased following treatment with 5-Aza-dC, DZNep, or TSA alone (Figure 7B, lanes 2, 4, and 5), as well as by combined treatment with DZNep and TSA (Figure 7B, lane 8). The number of cells in the G2/M-phase were also increased by combined treatment with RG108 and TSA or DZNep, and TSA (Figure 7C, lanes 7 and 8). In HSC-2 cells, treatment with 5-Aza-dC or RG108 alone (Figure 7E, lanes 2 and 3), or a combination of 5-Aza-dC or DZNep and TSA (Figure 7E, lanes 6 and 8) increased the number of cells in the S-phase. Treatment with 5-Aza-dC alone, or combined with TSA, also caused significantly more cells to be in the G2/M-phase (Figure 7F, lanes 2 and 6), which was accompanied by a decrease in the number of cells in the G0/G1 phase (Figure 7D, lanes 2 and 6). These results indicate that DNMTi, HMTi, and HDACi treatment alone, or in combination, induce cell cycle arrest in the S-phase and G2/M-phase in Ca9-22 and HSC-2 cells.

### 3.7. Histone H2AX Phosphorylation in OSCC Cells Increases after Treatment with DNMTis, HMTis, and HDACis

**Figure 8:**
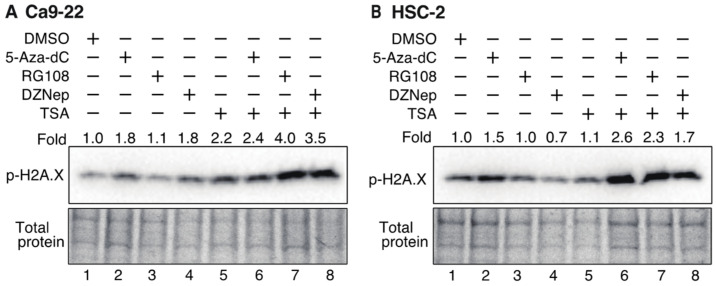
Phosphorylation of the histone variant H2A.X in Ca9-22 and HSC-2 cells treated with DNMTis, HMTis, and HDACis. p-H2A.X levels in Ca9-22 (**A**) or HSC-2 (**B**) following treatment with 5-Aza-dC (0.1 µM), RG108 (5 µM), DZNep (0.2 µM), or TSA (0.05 µM) alone or in combination for 72 h. p-H2A.X levels were normalized to the total loading protein. The fold expression of p-H2A.X relative to DMSO-treated samples is shown above each lane in the blots.

To determine whether DNA damage is involved in cell cycle arrest, phosphorylation of the histone variant H2A.X (γH2A.X), which is an indicator of double-stranded DNA breaks [52], was examined by Western blotting (Figure 8). Ca9-22 and HSC-2 cells were treated with various inhibitors for 72 h and γH2A.X expression was then detected using an anti-phosphorylated H2A.X antibody. In Ca9-22 cells, treatment with 5-Aza-dC, DZNep, and TSA alone caused an approximate 2-fold increase in γH2A.X (Figure 8A, lanes 2, 4, and 5), while combined treatment with 5-Aza-dC and TSA, RG108 and TSA, or DZNep and TSA further enhanced phosphorylation (Figure 8A, lanes 6, 7, and 8). In HSC-2 cells, treatment with 5-Aza-dC alone increased γH2A.X by 1.5-fold (Figure 8B, lane 2) while combined treatment with 5-Aza-dC and TSA, RG108 and TSA, or DZNep and TSA caused additional γH2A.X expression (Figure 8B, lanes 6, 7, and 8). These results indicate that combined treatment with DNMTis, HMTis, and HDACis causes increased double-stranded DNA breaks and enhances γH2A.X expression.

Taken together, these results indicate that the combined treatment with DNMTis, HMTis, and HDACis decreases Ca9-22 and HSC-2 cell viability via apoptosis and cell cycle arrest.

## 4. Discussion

Epigenomic abnormalities such as DNA methylation, histone methylation, and acetylation are involved in carcinogenesis. Thus, epigenome-targeted pharmacotherapy has been applied clinically [4]. However, the efficacy of epigenomic therapy for OSCC and the underlying molecular mechanisms have not been fully elucidated. In this study, we investigated the effect of combined treatment with low DNMTi, HMTi, and HDACi concentrations on OSCC cell viability and the associated molecular mechanism. Combined treatment with 5-Aza-dC, RG108, and TSA significantly decreases Ca9-22 and HSC-2 cell viability. In Ca9-22 cells, combined treatment with DZNep, HMTi, and TSA also decreased viability. Our results also indicate that the molecular mechanism associated with this decrease in cell viability after combined treatment is mediated by apoptosis and cell cycle arrest in the S and G2/M phases due to DNA damage.

Decreased cell viability is enhanced by DNMTis and HDACis in various cancer cell lines [35,36,37,38,39,40]. The underlying molecular mechanisms include the reactivation of epigenetically repressed tumor suppressor genes and apoptosis-related genes [35,38,39,40], as well as the mechanisms independent of induced gene expression [53]. Methyl-CpG-binding protein (MeCP2) binds to methylated DNA regions and forms a complex with HDACs to deacetylate histones, further enhancing the transcriptional repressive state [5,20]. The robust repression of gene expression by DNA methylation and histone deacetylation is reactivated by TSA and 5-Asa-dC, by inducing the expression of tumor suppressor genes, cell growth regulator genes, and apoptosis-related genes [38,39,40]. However, the decreased cell viability caused by combined treatment with DNMTi and HDACi is also enhanced by DNA damage [53]. HDACs also contribute to the regulation of non-histone protein expression, such as ataxia-telangiectasia mutated (ATM), which is also involved in DNA replication and DNA damage repair [54]. HDACs are upregulated in various cancer cells [24]. Thus, inhibiting HDACs results in downregulated DNA repair genes and suppressed DNA damage repair, leading to cell cycle arrest and cell death in cancer cells [22]. Hence, when DNA damage is induced by 5-Aza-dC [55,56], an insufficient DNA repair response occurs due to decreased expression of DNA repair genes by HDACis, resulting in persistent DNA damage. In the present study, the combined treatment with DNMTis and HDACis enhanced γH2A.X expression, indicating double-stranded DNA breakage [52], resulting in cell cycle arrest in the S and G2/M phases.

We also observed that combined treatment with DZNep and TSA significantly decreased Ca9-22 cell viability. DZNep suppresses histone methyltransferase EZH2, a component of polycomb repressor complex 2 (PRC2), and inhibits H3K27 trimethylation, a repressive histone code for gene expression (H3K27me3) [15]. In colorectal cancer cells, combined treatment with DZNep and TSA decreases H3K27me3 and increases trimethylation of the activating histone code, H3K4 (H3K4me3) [57], which causes a repressive chromatin state to convert into an active state and induces major changes in gene expression [57]. In breast cancer cells and acute myeloid leukemia cells, combined treatment with DZNep and TSA induces cell cycle arrest in the G2/M-phase and causes apoptosis [58,59]. In the present study, combined treatment with DZNep and TSA in Ca9-22 cells induced enhanced caspase 3/7 activity and cell cycle arrest in the S and G2/M phases. However, the molecular mechanisms underlying caspase activation and cell cycle arrest remain to be determined.

We also show that treatment with 5-Aza-dC alone induces a concentration-dependent decrease in cell viability. The decreased cell viability from 5-Aza-dC appears to be due to a mechanism independent of DNA demethylation. The 5-Aza-dC, a nucleoside analog, is incorporated into the DNA strand during DNA replication and binds irreversibly to DNMT1, thereby inhibiting its activity and inducing DNA demethylation [7]. Incorporated 5-Aza-dC inhibits DNA replication and induces DNA damage. In the present study, γH2A.X expression was observed in Ca9-22 and HSC-2 cells after treatment with 5-Aza-dC alone, suggesting that 5-Aza-dC induces double-strand DNA breaks.

RG108 is a DNMTi designed to inhibit the active site of DNMT1 and has low cytotoxicity in colorectal cancer cells and promyelocytic leukemia cells [8,60]. In this study, RG108 alone did not induce a decrease in OSCC cell viability after 72 h. However, combined treatment with RG108 and TSA decreased cell viability and enhanced γH2A.X expression, suggesting that the growth inhibition was due to cell cycle arrest caused by double-stranded DNA breaks. Sensitivity to RG108 appears to vary in different cancer cell types. For instance, RG108 treatment alone induces decreased cell viability, increased radiosensitivity, and cell cycle arrest in the G2/M-phase of endometrial carcinoma and esophageal squamous cell carcinoma cells [61,62]. The differential sensitivity of cancer cells to RG108 may depend on the methylation status of centromeres and euchromatin regions that are involved in maintaining chromosome stability [8].

An intriguing observation of this study is that the sensitivity to epigenomic drugs differs between Ca9-22 and HSC-2 cells. In Ca9-22 cells, treatment with TSA alone, or in combination with DZNep and TSA, significantly decreases cell viability and enhances caspase 3/7 activity. In contrast, HSC-2 cells were less sensitive to TSA, and neither activation of caspase 3/7 nor decreased cell viability was observed after combined treatment with DZNep and TSA. Although we did not investigate the molecular basis for this difference in sensitivity, there are several possible mechanisms. First, differences in the expression of multidrug resistance proteins (MRPs), such as ABC transporters [63], may contribute to the differential sensitivity to epigenomic drugs. However, the differential expression of these MRPs in HSC-2 and Ca9-22 cells, and their effects on the epigenomic drugs used in this study, have not been reported. Second, differences in constitutive activation of nuclear factor-κB (NF-κB), a transcription factor that controls the expression of genes involved in survival and growth of cells, may influence the observed differences in sensitivity [64,65]. NF-κB is constitutively activated in many cancer cells and is involved in resistance to chemotherapy and radiotherapy by altering the gene expression involved in cell survival and proliferation [66]. Indeed, HSC-2 cells exhibit constitutive NF-κB activation [64], which may contribute to the decreased sensitivity to TSA. Third, differences in mutations of the tumor suppressor gene p53 (*TP53*) may impact drug sensitivity [67]. p53 regulates cell cycle progression, DNA repair, and cell death, and suppresses malignant transformation of cells [68]. *TP53* is frequently mutated in OSCCs [69]. In fact, these mutations have also been reported in Ca9-22 and HSC-2 cells [69] and include loss-of-function and gain-of-function mutations [70,71]. Within HSC-2 cells, these mutations are caused by point mutations (G>A) of the splicing donor at the boundary of exon 6 and intron 6, resulting in a frameshift and the appearance of a termination codon in exon 7 [69]. In preliminary experiments, we confirmed that p53 protein expression was not observed in HSC-2 cells (data not shown). In contrast, the *TP53* mutation in Ca9-22 cells is caused by a missense mutation in the DNA-binding domain, which is a hot spot for *TP53* mutations. This change converts arginine 248 (R) to tryptophan (W), resulting in the production of a full-length mutant p53 (R248W) [69] that retains DNA-binding activity and protein-protein interactions [71]. Therefore, the induced apoptosis in Ca9-22 cells after combined treatment with DZNep and TSA may be due to the increased apoptosis-related gene expression associated with the gain-of-function mutant p53. Additionally, acute myeloid leukemia cells treated with 5-Aza-dC combined with an HDACi show caspase 3 activation in leukemia cells with the p53R428W mutation [72]. The sensitivity of cancer cells to HDACis is higher in cells with p53R428W mutations than in cells with a loss of p53 [73], which is consistent with the difference in sensitivity to TSA between Ca9-22 and HSC-2 cells in the present study. Therefore, when considering epigenomic therapy for OSCC, it is important to analyze the nature of constitutive NF-κB activation and *TP53* mutation. Further studies are needed to understand the molecular mechanisms underlying the different sensitivities of Ca9-22 and HSC-2 cells to epigenomic drugs.

The key findings of the current study include (i) demonstrating the efficacy of combination treatment with low concentrations of epigenomic drugs on OSCC cells; (ii) partially elucidating the molecular mechanism associated with the anticancer effects of combined treatment. Nevertheless, certain limitations were noted in the current study. First, this study only demonstrated the cytotoxic effects of epigenomic drugs in a two-dimensional (2D) cell culture model. Thus, if clinical significance is to be achieved, in vivo preclinical studies in mice are needed. Moreover, three-dimensional (3D) cell culture models, such as spheroids and organoids, have recently been characterized as more physiologically representative of the human tumor microenvironment than conventional 2D cell culture models [74,75]. Therefore, future research should also include investigation of the combined epigenomic drug treatment using three-dimensional (3D) cell culture models as a bridge between two-dimensional (2D) cell culture and animal models. Second, investigations on HDACs and DNMTs expression levels, as well as the gene expression pattern, following the treatment with combined HDACi, DNMTi, and HMTi, are necessary to clarify the mechanisms by which these inhibitors induce antiproliferative and pro-apoptotic effects on the OSCC cells used in this study.

Surgical resection of OSCCs may cause deterioration of the quality of life of the patient, including disrupted facial appearance and dysphagia. Recently, immunotherapy with immune checkpoint inhibitors targeting programmed cell death protein-1/2 (PD-1, PD-2) and cytotoxic T lymphocyte-associated antigen-4 (CTLA-4) has been clinically applied as a fourth therapeutic modality [76]. However, while these immunotherapies show high antitumor efficacy in some patients, others do not experience therapeutic benefit [77,78]. One of the reasons for this non-responsiveness depends on epigenomic abnormalities [77]. Preclinical studies showed that combined epigenomic drugs enhance the response to immunotherapy [79]. Our study demonstrates that the combination of low concentrations of epigenetic agents is effective for OSCCs. Thus, our findings advance the current understanding of the molecular mechanism associated with combined immunotherapy and epigenetic therapy.

## Data Availability

The data presented in this study are available in this article.
